# Nitric Acid and Opium in Diarrhœa and Dysentery

**Published:** 1861-12

**Authors:** 


### NITRIC ACID AND OPIUM IN DIARRHŒA AND DYSENTERY.

Dr. Hynes, in a communication to the *Lancet*, recommends the following formula, as one he has found highly beneficial in the autumnal forms of diarrhœa and dysentery:—

Compound infusion of gentian, eight ounces; tincture of opium, a drachm to a drachm and a half; nitric acid, twenty minims; one ounce to be taken after every liquid stool or painful alvine evacuation. A mustard plaster applied to the epigastrium, and drinking sparingly of ice-cold mint-tea, relieve the sickness and thirst that frequently accompany the severer form of these diseases.

Another formula is given by Mr. Hope, of Chatham, as follows:—

Nitric acid, two drachms; opium, two grains; water, two ounces; a spoonful to be taken in any vehicle three or four times daily.

Dr. Hynes adds, "I am unwilling to theorize upon the *methodus medendi* of what I believe to be the principal agent in the above formula; yet I cannot help thinking that the nitric acid possesses some disinfecting agency, no less than an astringent efficacy, over autumnal diseases. 'The fumes of nitric acid are believed to be efficacious,' says the late Dr. Montgomery, 'in destroying the effluvia of typhus and other febrile diseases.' Diluted with water, so as to form an acidulous drink, Dr. Duncan, of Edinburgh, used to employ it in the low fevers that prevailed in the suburbs of that town. But independently of its chemical action over animal effluvia, it appears to me to act as a direct astringent in all diseases of the mucous membrane. Thus in purulent ophthalmia, what remedy is so efficacious as nitrate of silver in solution? In fact, in all mucous discharges it is almost the sole remedy upon which the surgeon depends; and of course the nitric acid is the chief agent in this valuable therapeutic. I am in the habit of employing a formula of a nearly similar kind as a topical application, but with double the amount of acid, in cynanche and in diphtheria; and I can speak very strongly upon its beneficial effects. In broken-down constitutions impaired by mercury, by syphilis or other irregularities, the above remedy will be found frequently valuable; and given in combination with taraxacum, it will prove very serviceable in sluggish conditions of the liver. I am in the habit of employing it with very considerable advantage in the diarrhœa of infants, and, combined with the muriated tincture of iron, in tabes mesenterica. I may further state that I have given the other acid, singly and in combination, the full benefit of a fair trial in all the above forms of diseases; and, without any prepossession or prejudice, my experience enables me to give a decided preference to the claims of the nitric acid in combination with opium, as a very superior therapeutic remedy."

